# Host iron redistribution as a risk factor for incident tuberculosis in HIV infection: an 11-year retrospective cohort study

**DOI:** 10.1186/1471-2334-13-48

**Published:** 2013-01-29

**Authors:** Joann M McDermid, Branwen J Hennig, Marianne van der Sande, Adrian VS Hill, Hilton C Whittle, Assan Jaye, Andrew M Prentice

**Affiliations:** 1 Division of Nutritional Sciences, Cornell University, 310 Savage Hall, Ithaca, NY, 14853, USA; 2 Medical Research Council International Nutrition Group, Faculty of Epidemiology & Population Health, London School of Hygiene & Tropical Medicine, London, United Kingdom and Medical Research Council, Keneba, The Gambia; 3 Epidemiology and Surveillance Unit, Centre of Infectious Disease Control, National Institute of Public Health and the Environment, Bilthoven, The Netherlands and Julius Center, University Medical Center Utrecht, Utrecht, The Netherlands; 4 Wellcome Trust Centre for Human Genetics, University of Oxford, Oxford, United Kingdom; 5 Medical Research Council Laboratories, Fajara, The Gambia

## Abstract

**Background:**

Identifying people at higher risk of developing tuberculosis with human immunodeficiency virus (HIV) infection may improve clinical management of co-infections. Iron influences tuberculosis (TB) pathogenesis, but understanding the exact mechanisms of how and timing of when iron is involved remains challenging since biological samples are rarely available from the disease susceptibility period due to the difficulty in predicting in who and when, if ever, TB will develop. The objective of this research was to determine how host iron status measured at HIV diagnosis and genotypes related to host iron metabolism were associated with incident TB.

**Methods:**

Archived clinical data, plasma and DNA were analyzed from 1139 adult participants in a large HIV-1, HIV-2 and dual seroprevalent cohort based at the Medical Research Council Laboratories in The Gambia. Incident pulmonary and/or extrapulmonary TB diagnoses a minimum of 28 days after HIV diagnosis were independently re-confirmed using available evidence (n=152). Multiple host iron status biomarkers, Haptoglobin and solute carrier family 11, member 1 (SLC11A1) genotypes were modeled to characterize how indicators of host iron metabolism were associated with TB susceptibility.

**Results:**

Hemoglobin (incidence rate ratio, IRR=0.88, 95% CI=0.79-0.98), plasma transferrin (IRR=0.53, 0.33-0.84) and ferritin (IRR=1.26, 1.05-1.51) were significantly associated with TB after adjusting for TB susceptibility factors. While genotype associations were not statistically significant, SLC11A1 associations replicated similar directions as reported in HIV-seronegative meta-analyses.

**Conclusions:**

Evidence of host iron redistribution at HIV diagnosis was associated with incident TB, and genetic influences on iron homeostasis may be involved. Low hemoglobin was associated with subsequent diagnosis of TB, but when considered in combination with additional iron status biomarkers, the collective findings point to a mechanism whereby anemia and iron redistribution are likely due to viral and/or bacteria-driven processes and the host immune response to infection. As a result, iron supplementation may not be efficacious or safe under these circumstances. Clinical and nutritional management of HIV and Mycobacterium tuberculosis co-infected individuals, especially in regions where food insecurity and malnutrition co-exist, may be further improved when the iron-related TB risk factors identified here are better understood and managed to favor host rather than pathogen outcomes.

## Background

In 2011, an estimated 8.7 million new cases of tuberculosis (TB) arose from the approximately 1/3 of the world’s population infected with *Mycobacterium tuberculosis* (*Mtb*) [1]. This is an enormous disease burden, but it also underscores that infection with *Mtb* alone is insufficient to cause TB disease since the vast majority successfully contain their infection as indicated by the cumulative lifetime TB risk approximated between 5 to 10% in the absence of human immunodeficiency virus (HIV) co-infection [2]. Among the subgroup of *Mtb*-infected individuals that do progress to disease rapidly after initial infection or after a long period of clinical latency, there are dynamic and complex interactions that occur between multiple host and pathogen factors that must ultimately tip the balance towards disease occurrence. While some risk factors like HIV co-infection are well recognized, other risk factors like nutritional status of the host (or pathogen) have thus far been poorly characterized.

Like the human host and other microbes, *Mtb* has developed an intricate system of acquiring, metabolizing and storing essential iron under host-imposed states of iron deficiency and excess. Despite host attempts to restrict available iron to the microbe, *Mtb* is very successful in accessing multiple host iron sources including lactoferrin, ferritin and transferrin (Tf) [3], and recent evidence has revealed iron can also be obtained via a heme-acquisition system [4,5]. Host mechanisms also thwart bacterial iron acquisition and examples include host siderocalin binding to iron-laden *Mtb* siderophores that have captured host iron [6], plasma haptoglobin (Hp) binding free hemoglobin (Hb) to reduce host peripheral iron availability [7], increasing hepcidin-mediated ferroportin degradation to impair host iron efflux from intestinal enterocytes and macrophages [8], and altering host macrophage iron concentrations via the solute carrier 11 family of genes that encode proteins involved in phagolysosomal iron transport [9].

Host iron status and iron trafficking between the host and pathogen may affect the critical host-pathogen battle for essential iron, and thereby influence the development of TB. We hypothesized that the risk of TB in people living with HIV infection increases with elevated host iron status, increased macrophage iron availability and suboptimal regulation of iron distribution (Additional file 1). The primary research objective of this study was to describe how host iron status at HIV diagnosis indicated by plasma iron status biomarkers and iron-genes, including solute carrier family 11 (proton-coupled divalent metal ion transporters), member 1 (*SLC11A1*) operating at the level of the macrophage, were associated with the later development of TB in HIV infection. The main purpose of this study was to understand which iron status biomarkers were associated with TB risk in HIV infection in order to further the understanding of biomarker profiles that predict TB risk, and to guide evidence-based nutritional and clinical management of iron status among people with HIV infection who are also at risk of developing TB. To our knowledge, *Haptoglobin* genotypes have not been reported in association with incident TB in HIV infection, and *SLC11A1* genotypes have been restricted primarily to investigating TB susceptibility among HIV-seronegative participants. This research demonstrates that iron redistribution at the time of HIV diagnosis is associated with a significantly greater probability of developing TB, and *SLC11A1* genotypes may influence TB susceptibility in individuals with HIV co-infection like has been demonstrated among HIV-negative individuals [10].

## Methods

### Study setting

Archived data and biological samples were obtained from the HIV Clinical Cohort based at The Medical Research Council (MRC) Laboratories in The Gambia. The first HIV infection was detected in The Gambia in 1986, and this prospective cohort was established shortly thereafter to study the emerging HIV-1, HIV-2 and HIV-dual infection epidemics unique to this region. Cohort participants were recruited nation-wide and all were offered free clinical care according to Gambian national guidelines in effect at the time of their participation, including co-trimoxazole prophylaxis and symptom management. For the participants included in the current study, anti-retroviral therapy and viral load data were unavailable at that time.

### Study design

Eligibility for this retrospective cohort study included all HIV Clinical Cohort participants who: i) were ≥18 years; ii) had an archived baseline plasma sample stored at –20 to –80°C and collected within 90 days of cohort entry and additionally, if they were diagnosed with incident TB, if their plasma sample was taken >28 days before TB diagnosis; and iii) had an archived buffy coat or PBMC sample stored at –80°C or in liquid nitrogen that was available for DNA extraction. To establish incident TB cases for this study, two physicians supervised the re-examination of all original clinical, microbiological and radiographic evidence for cohort participants who had a provisional TB diagnoses recorded between 01/01/1991-31/12/2001 in order to confirm the original TB diagnosis. First instances of the following were classified as incident TB: confirmed pulmonary TB (PTB) defined by the presence of acid-fast bacilli in direct smear or culture from sputum or lavage, or smear-negative PTB defined by the presence of strongly suggestive clinical symptoms and radiographic signs consistent with PTB; and/or confirmed extra-pulmonary TB (EPTB) defined by the demonstration of acid-fast bacilli in a biopsy or aspirate of a lymph node, or any other normally sterile site by smear or culture, or probable EPTB defined by strongly suggestive clinical features of EPTB. Participants with prevalent disease (i.e. any form of TB diagnosed ≤28 days following HIV diagnosis) were excluded from this study. Cohort follow-up of participants occurred at regularly scheduled clinic visits, or when participants presented for clinical care at any time. If participants failed to return to the clinic, fieldworkers visited their residence throughout the country to ascertain their mortality status. Censoring of data occurred when one of the mutually exclusive events occurred: end of the study follow-up period; first instance of TB; death or loss to follow-up defined as the last date known with certainty to be alive.

### Laboratory protocols

Details of iron and candidate gene analyses have been previously published [11]. Briefly, soluble transferrin receptor (sTfR) (R&D Systems, Abingdon, UK) and ferritin (Immuno-biological Laboratories, Hamburg, Germany) were measured by enzyme-linked immune-sorbent assay (ELISA). Plasma iron was assessed using an endpoint assay (ABX Diagnostics, Shefford, UK) and Tf by turbidimetry (ABX Diagnostics, Shefford, UK). Hemoglobin (Hb) concentrations were measured during routine clinical screening and obtained from study databases. Alpha-_1_-antichymotrypsin (ACT) was measured using a nephelometric assay (DakoCytomation Inc, Ely, UK).

DNA extraction occurred at the MRC Laboratories in The Gambia, and all genotyping was performed at the Wellcome Trust Centre for Human Genetics in Oxford. *HP* genotyping was performed using an allele-specific polymerase chain reaction (PCR)–based method distinguishing the major allelic variants (Hp1 and Hp2*)* of the *α*-chain [12]. *SLC11A1* polymorphisms SLC1 (rs34448891) and CAAA (rs17229009) were genotyped by fluorescent polyacrylamide gel electrophoresis of amplicons on an ABI 3700 sequencer (Perkin Elmer-ABI, Foster City, USA). SLC3 (rs3731865), SLC6a (rs17235409) and SLC6b (rs17235416) were genotyped using the Sequenom platform (hME Sequenom, http://www.sequenom.com). Internal control samples were included during genotyping for quality control.

### Statistical analysis

The primary outcome was incident TB, with main effects for iron status or iron-metabolism genotypes assessed using complete data with multiplicative Poisson regression models and differences estimated on a log scale and expressed as incidence rate ratios (IRR) (Stata MP 11.2, Statacorp, Texas, USA). Potential confounders considered *a priori* were HIV-type, sex, self-reported ethnicity (for genotype associations) and baseline absolute CD4 cell count, age, body mass index (BMI) [13] and ACT concentrations. Genotype data was assessed based on dominant models, and adjustments were not made for multiple testing as the genotypes selected here were based on *a prior* hypotheses rather than data-driven or post-hoc analyses. Missing data on potential confounders (self-reported ethnicity, hemoglobin, BMI, CD4) and genotyping failures for some subjects influenced the ability to detect statistical significance in regression models that included these variables. Sensitivity analyses were conducted including microbiologically-confirmed cases of TB only versus all TB cases that included a clinical diagnosis, and as the overall interpretation was not altered all TB cases were included in all analyses.

### Ethics considerations

Ethical approval for this study was granted by the ethical committees of the Joint Gambian Government/MRC Laboratories, the London School of Hygiene & Tropical Medicine and Cornell University. All participants provided voluntary written informed consent when enrolling in the main MRC HIV Clinical Cohort and the original consent form used was included as part of the ethical review for this study.

## Results

### Incident TB

**Figure 1:**
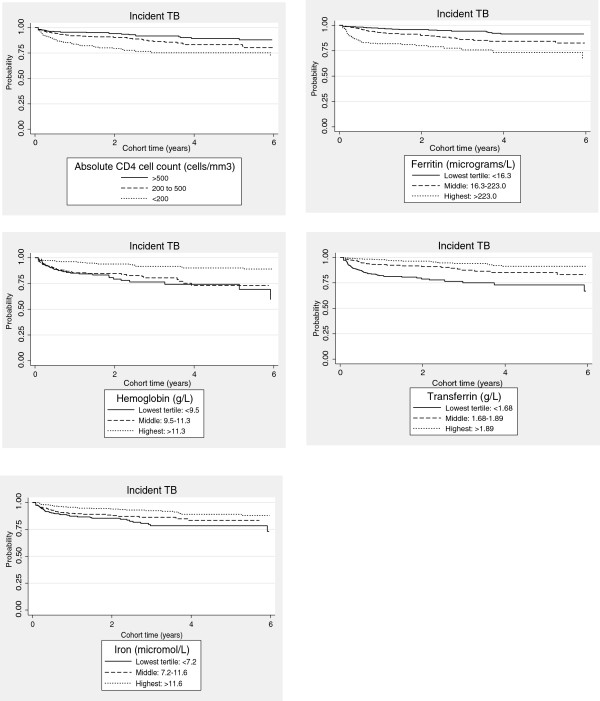
Risk of incident tuberculosis in HIV according to absolute CD4 cell count and iron biomarkers.

Of the 1139 individuals eligible for this study, the median time-to-TB diagnosis following cohort entry at first HIV diagnosis was approximately six months (interquartile range = 4 to 8 months), with microbiologically-confirmed PTB the most common TB diagnosis (n = 107/152 incident TB cases) (Table 1). Demographic and clinical characteristics are summarized in Additional file 2. Females (unadjusted IRR, 95% CI = 0.53, 0.38 to 0.73) and participants with higher CD4 (unadjusted IRR, 95% CI = 0.56, 0.50 to 0.64) were less likely to develop incident TB, while those with a BMI < 18.5 kg/m^2^ were more likely to develop TB (unadjusted IRR, 95% CI = 2.02, 1.33 to 3.08). In Figure 1, CD4 counts categorized according to clinical categories (i.e. > 500, 200-500, < 200 cells/mm^3^) provide clear evidence that, as expected, greater immunosuppression was associated with an increased likelihood of developing TB (*P* < 0.001).

**Table 1:** Cohort follow-up and frequency of tuberculosis diagnoses

**Number of cohort participants**	**1139**		
**Person-years of follow-up**	**2708**		
**Median days to incident tuberculosis diagnosis**, **interquartile range**	**173(114-237)**		
***Tuberculosis diagnoses***	***Number of incident tuberculosis cases***	***Percentage of all incident tuberculosis casescxx***	***Percentage of HIV Clinical Cohort participants in this study***
Tuberculosis, all cases	152	100	13
Pulmonary, all cases	135	89	12
Pulmonary, culture-confirmed	27	18	2
Pulmonary, sputum-positive	80	53	7
Pulmonary, sputum-negative	44	26	4
Extra-pulmonary tuberculosis, all cases	23	15	2
Extra-pulmonary, culture-confirmed	13	9	1
Extra-pulmonary, probable	10	7	1

^*^Pulmonary and extra-pulmonary tuberculosis classifications were not mutually exclusive, therefore percentage totals may not sum to 100%.

### Iron redistribution is a risk factor for the development of TB in HIV infection

At baseline, participants that subsequently developed TB had lower Tf and iron, and higher ferritin concentrations than participants who did not develop TB (Additional file 2). Kaplan-Meier curves graphically indicated that this association was in a dose-response manner and statistically significant for Hb, Tf, iron and ferritin tertile categories (In Figure 1; all *P* < 0.001). Univariate regression analyses (Table 2, Models 1) indicated inverse associations for Hb, iron and Tf concentrations (i.e. for each one unit increase in concentration, there was a significantly reduced probability of TB), while increased ferritin concentrations were directly associated with incident TB (i.e. for each one unit increase in concentration, there was a significantly increased probability of TB). Neither sTfR nor transferrin saturation were associated with incident TB diagnosis. In Table 2, Models 2, adjusting for the acute phase response (APR) assessed by ACT concentration only slightly modified the magnitude of observed effects compared to the unadjusted models, and did not alter the overall interpretation. Likewise, even after adjusting for several known risk factors for TB (BMI, absolute CD4 cell count, age, sex: Table 2, Models 3), Tf, ferritin and Hb concentrations remained significantly associated with the development of TB in HIV infection.

**Table 2:** Host iron as a risk factor for incident tuberculosis in HIV infection

	**Model 1: IRR, unadjusted**		**Model 2: IRR, adjusted for acute phase response**		Model 3: IRR, adjusted for known TB risk factors*	
	**Is the host iron status biomarker a risk factor for incident TB in HIV infection?**		**Does the biomarker remain an independent risk factor after adjusting for a marker of the host acute phase response (baseline α-**_**1**_**-antichymotrypsin concentration)?**		**Does the biomarker remain an independent risk factor for incident TB in HIV infection after adjusting for baseline age, CD4, body mass index?**	
**Iron factors**	**IRR (95% CI)**	***P***	**IRR (95% CI)**	***P***	**IRR (95% CI)**	***P***
Transferrin, g/L	0.25 (0.19-0.35)	< 0.001	0.30 (0.21-0.43)	< 0.001	0.53 (0.33-0.84)	0.007
Transferrin saturation, %	1.00 (0.99-1.01)	0.691	1.00 (0.99-1.01)	0.691	1.00 (0.99-1.01)	0.669
Ferritin, μg/L	1.71 (1.49-1.97)	< 0.001	1.60 (1.35-1.89)	< 0.001	1.26 (1.05-1.51)	0.014
Hemoglobin, g/L	0.79 (0.73-0.86)	< 0.001	0.84 (0.77-0.92)	< 0.001	0.88 (0.79-0.98)	0.023
Iron, μmol/L	0.92 (0.88-0.95)	< 0.001	0.94 (0.91-0.97)	< 0.001	0.96 (0.92-1.01)	0.104
Transferrin receptor, nmol/L	1.01 (1.00-1.02)	0.160	1.01 (1.00-1.02)	0.130	1.00 (0.99-1.01)	0.991

Main effects for iron status biomarkers assessed using complete data with multiplicative Poisson regression models with differences estimated on a log scale and expressed as incidence rate ratios (IRR).

Ferritin and absolute CD4 cell counts were natural-logarithm transformed; BMI was dummy-coded as <18.5 or ≥18.5 kg/m^2^; self-reported ethnicity was dummy-coded as Mandinka, Wolof, Fula, Jola or Otherwise.

*In this dataset, unadjusted Poisson regression analyses indicated sex (females) (IRR, 95% CI = 0.53, 0.38 to 0.73), and higher absolute CD4 (IRR, 95% CI = 0.56, 0.50 to 0.64) were associated with significantly lower risk of incident TB and body mass index < 18.5 (IRR, 95% CI = 2.02, 1.33 to 3.08) was associated with a significantly higher risk of TB. Age (IRR, 95% CI = 1.02, 1.00 to 1.04) was not a direct risk factor for TB in this dataset, but age was included in models as it is commonly associated with differential risk of TB.

CI = 95% confidence interval; IRR = incidence rate ratio; TB = tuberculosis (all forms).

### Iron-related genotypes and the development of TB in HIV infection

Regression analyses of unadjusted and adjusted models of iron-metabolism genotypes were not statistically significant (Table 3). In Table 3 Models 2, adjusting for a number of known risk factors for TB (baseline BMI, absolute CD4 cell count, age, sex) considerably modified the magnitude of effect of carriage of the TB predisposing minor allele for SLC1 (rs34448891) and SLC3 (rs3731865), however, these associations were not statistically significant.

**Table 3:** Host iron genotypes as a risk factor for incident tuberculosis in HIV infection

***Iron genotypes***	**Model 1: IRR, unadjusted**		Model 2: IRR, adjusted for known risk factors for TB*	
	**Is the iron genotype a risk factor for incident TB in HIV infection?**		**Does the iron genotype remain a risk factor for incident TB after adjusting for sex, baseline age, CD4, body mass index plus self-reported ethnicity?**	
Haptoglobin*				
Hp 1-1	Reference		Reference	
Hp 2-1, Hp 2-2	1.03 (0.68-1.56)	0.879	0.83 (0.47-1.49)	0.525
*SLCA11A1*				
SLC1 (rs34448891) Allele 3/Allele 3	Reference		Reference	
Allele 3/Other, Other/Other	0.96 (0.65-1.42)	0.832	1.29 (0.73-2.29)	0.377
SLC3 (rs3731865) G/G	Reference		Reference	
C/G, C/C	1.05 (0.65-1.69)	0.853	1.56 (0.85-2.87)	0.151
SLC6a (rs17235409) G/G	Reference		Reference	
A/G, A/A	0.95 (0.54-1.68)	0.869	1.10 (0.51-2.36)	0.813
SLC6b (rs17235416) TGTG +/+	Reference		Reference	
TGTG +/-, -/-	0.90 (0.59-1.36)	0.610	1.00 (0.56-1.78)	0.996
CAAA (rs17229009) CAAA+/+, +/-	Reference		Reference	
CAAA -/-	0.78 (0.49-1.25)	0.302	0.85 (0.45-1.61)	0.615

Main effects for iron-metabolism genotypes assessed using complete data with multiplicative Poisson regression models with differences estimated on a log scale and expressed as incidence rate ratios (IRR).

Absolute CD4 cell counts were natural-logarithm transformed; BMI was dummy-coded as <18.5 or ≥18.5 kg/m^2^; self-reported ethnicity was dummy-coded as Mandinka, Wolof, Fula, Jola or Otherwise.

*In this dataset, unadjusted Poisson regression analyses indicated sex (females) (IRR, 95% CI = 0.53, 0.38 to 0.73), and higher absolute CD4 (IRR, 95% CI = 0.56, 0.50 to 0.64) were associated with significantly lower risk of incident TB and body mass index < 18.5 (IRR, 95% CI = 2.02, 1.33 to 3.08) was associated with a higher risk of TB. Age (IRR, 95% CI = 1.02, 1.00 to 1.04) was not a direct risk factor for TB in this dataset, but age was forced in models as it is commonly associated with differential risk of TB in the literature.

CI = 95% confidence interval; IRR = incidence rate ratio; TB = tuberculosis (all forms).

## Discussion

This study provides evidence that iron status measured by biomarkers associated with distinct roles in iron metabolism and homeostasis are risk factors for developing incident TB in immunocompromised HIV-infected individuals. To our knowledge, this data demonstrates for the first time in a human cohort study the temporal association whereby iron status assessed prior to TB diagnosis is associated with susceptibility to TB, rather than the more commonly studied associations reported at TB diagnosis or in relation to post-TB diagnosis clinical outcomes [14-17]. The pattern of iron status biomarkers we observed is characteristic of iron redistribution occurring with anemia of chronic disease/anemia of inflammation. This suggests a complicated mechanistic process whereby TB risk may be related to differential host iron handling and/or bacterial iron availability in the subgroup of people with *Mtb* and HIV co-infections who are susceptible to TB.

An iron-related risk profile observed in this study is characteristic of anemia of inflammation mediated by the APR, and since all cohort participants were HIV-seropositive and living with a chronic infection, most participants were experiencing varying degrees of APR. During the APR, inflammatory cytokines mediate the redistribution of body iron from the systemic circulation to macrophage iron stores, with resulting inhibition of erythropoiesis. Under conditions of APR, hepcidin would be rapidly induced through both interleukin (IL)-6 and bone morphogenetic protein dependent pathways resulting in the inhibition of ferroportin-mediated iron efflux from enterocytes and macrophages [18-20]. Overall, APR results in reduced dietary iron uptake, reduced erythropoeisis and, importantly, increased macrophage iron retention [21]. It is the consequences of this iron redistribution that may alter both the host innate immune response to infection [22] and *Mtb* virulence through an increased ability of *Mtb* to access increasingly available host iron sources. Macrophage iron concentration may favor intracellular pathogens, perhaps explaining *Mtb*’s niche environment within the macrophage. Although we have not measured plasma hepcidin concentrations or directly measured macrophage iron in this study, *in vitro* and animal model data indicates iron influences the development of TB and iron-loaded macrophages promote *Mycobacterium* growth [23-27]. Overall, our data provide support for a working hypothesis that host iron redistribution favors pathogen virulence at the dynamic and evolving host-pathogen interface. It is likely that each iron factor is associated with known and yet unknown roles in iron trafficking, metabolism and homeostasis and those iron-regulated/-catalyzed host immune defenses collectively change the risk of disease. It remains unanswered as to what degree managing iron redistribution during HIV infection, *Mtb* infection or co-infection leads to the development of TB.

Low Hb concentration and anemia (of unknown etiology) are frequently considered an indication of dietary iron insufficiency among people living in regions where malnutrition and food insecurity are common and treated using supplemental iron. While low Hb concentrations were associated with an increased risk of developing TB in this study, by interpreting low Hb in combination with the other iron and inflammatory markers it is more likely that anemia was due to reasons other than, or in addition to, dietary iron insufficiency alone. Similar conclusions were made in an Indonesian study by Sahiratmadja *et al.*[14] where despite the observation that most participants with TB had anemia of unknown etiology, spontaneous resolution of anemia was associated with TB treatment in the absence of iron supplementation. Likewise, Lee *et al.*[28] reported that anemia was common in Korean participants with TB, and similarly anemia resolved in 65% of participants receiving TB medication without iron replacement therapy, with the remainder having stable (26%) or improved Hb concentrations (9%) following TB treatment only.

Using an *a priori* candidate gene approach, we hypothesized that variation in the *HP* and *SLC11A1* genes would be associated with incident TB [29] (Additional file 1). While our data do not show statistically significant findings, the direction and magnitude of associations observed for *SLC11A1* are consistent with reports in African-Americans [30], South Africans [31] and those from a recent *SLC11A1* meta-analysis of studies across different ethnicities including HIV-negative participants from three African countries [10]. Li *et al.*[10] reported a significantly increased overall odds of TB was apparent for SLC1/rs34448891 [Odds ratio, (OR) Allele 3 versus Otherwise =1.31; 1.08 to 1.59], SLC3/rs3731865 (OR C/C plus C/G versus G/G =1.23; 1.05 to 1.44), SLC6a/rs17235409 (OR A/A plus A/G versus G/G =1.25; 1.04 to 1.50) and SLC6b/rs17235416 (OR TGTG -/- plus TGTG +/- versus TGTG +/+ =1.35; 1.17 to 1.54). In contrast, our SLC11A1 CAAA/rs17229009 results differed in direction to non-statistically significant results reported from a Malawian case-control study of HIV-seropositive and HIV-negative individuals [32]. A recent report has also presented evidence of a significant gene-gene interaction with *SLC11A1* and *IFNG1* among South Africans [33]. A number of Russian and Ukrainian studies have reported on Hp phenotypes and TB diagnosis or prognosis (reviewed in [29]) however, no differences in Hp phenotypes were observed between PTB cases and controls from Zimbabwe [34]. To our knowledge, however, this is the first study to report data for *HP* genotypes and TB susceptibility. Ultimately, as is the case for many individual genetic associations, studies, cumulative data from multiple studies will be important to unravel the true genetic associations and TB susceptibility, including the likely possibility of iron-gene interactions and that have been previously reported for HIV mortality from the MRC HIV Clinical Cohort [11].

The current study has strength in that the cohort design which allows temporal associations to be ascertained. However, it is possible that prevalent TB remained undetected at the time of blood sampling among some participants. This would likely have biased to the null, but overall it was likely to have a minimal impact since all participants received a full clinical consultation at the time of cohort entry and the median time to incident TB in was almost six months after enrolment. Despite the sensitivity analyses showing no differences in interpretation of models whether all TB cases or only proven TB cases were included, it may be possible that the associations we observed are only relevant to some forms of TB (i.e. PTB vs. EPTB). Although unavailable for this study, it would also be of interest to consider viral load in these associations.

## Conclusions

Our evidence suggests that iron redistribution reflecting a shift from the periphery to the macrophage storage site is a risk factor for incident TB among people with HIV co-infection. Given the pattern of iron redistribution evident in this study, it is unlikely that dietary iron insufficiency was the primary cause of low hemoglobin concentrations and therefore, supplemental iron to correct anemia would be unlikely to reduce the risk of developing TB. Identifying individuals at greater risk of TB using iron status biomarkers may lead to improved TB clinical and nutritional management, particularly for difficult to diagnose HIV and pediatric TB, and earlier diagnoses resulting in faster TB treatment may reduce TB transmission. Understanding host-*Mtb* interactions and the biological mechanisms underlying our observations may reveal exploitable targets for future pharmaceutical and nutritional interventions that will improve care of people living with *Mtb* and HIV co-infection who are at risk of developing TB.

## Abbreviations

ACT: Alpha-1-antichymotrypsin; APR: Acute phase response; BMI: Body mass index; ELISA: Enzyme-linked immune-sorbent assay; EPTB: Extra-pulmonary TB; HP/Hp: Haptoglobin/haptoglobin; Hb: Hemoglobin; HIV: Human immunodeficiency virus; IRR: Incidence rate ratio; MRC: Medical Research Council; *Mtb*: *Mycobacterium tuberculosis*; n: sample size; OR: Odds ratio; PCR: Polymerase chain reaction; PTB: Pulmonary tuberculosis; sTfR: soluble transferrin receptor; *SLC11A1*: Solute carrier family (proton-coupled divalent metal ion transporter) 11, member 1; Tf: Transferrin; TB: Tuberculosis.

## Competing interests

The authors declare they have no conflicts of interest. The project was funded in part by the Bristol Myers Squibb Mead Johnson Unrestricted Grant Programme, the Canadian Institutes of Health Research, the Medical Research Council and the Wellcome Trust.

## Authors’ contributions

JMM, MvdS, AVSH, HCW, AJ and AMP conceived of and designed the research; JMM conducted the plasma iron and ACT analyses, BJH conducted the genotyping and MvdS was responsible for the TB diagnoses; JMM analyzed the data and drafted the manuscript; all authors read and approved the final manuscript.

## Pre-publication history

The pre-publication history for this paper can be accessed here:

http://www.biomedcentral.com/1471-2334/13/48/prepub

## Supplementary Material

Additional file 1 Rationale for study hypothesis. Click here for file

Additional file 2 Baseline characteristics of participants. Click here for file
