# Supplementary material for: Host iron redistribution as a risk factor for incident tuberculosis in HIV infection: an 11-year retrospective cohort study
Source: BMC Infect Dis. 2013 Jan 29;13:48. doi: 10.1186/1471-2334-13-48 (PMC3568026; doi:10.1186/1471-2334-13-48)
Supplement: Additional file 1 — Rationale for study hypothesis. [file 1471-2334-13-48-S1.docx]

Additional File 1 Rationale for study hypothesis

|  | **Iron-related function, established** | **Biological mechanisms of effect, *a priori* hypothesized or evidence published** | **Direction associated with increased risk of tuberculosis, *a priori* hypothesized** |
| --- | --- | --- | --- |
| ***Iron at the level of host*** | | | |
| Plasma transferrin (Tf) | The Tf protein transports absorbed ferric iron from intestinal enterocytes to cell-associated transferrin receptor. This allows iron transfer from the extracellular to intracellular environments. | Known source of *Mycobacterium tuberculosis (Mtb)*-acquired host iron [3]. | ↓ (versus ↑) Tf concentration. Note: Counterintuitive, but when intracellular iron is elevated, Tf concentration is ↓. |
| Transferrin saturation (TS) | TS reflects the degree that Tf ferric-iron binding sites are occupied, with ↑ TS indicating ↑ quantity of occupied sites; calculated by (100 * plasma iron concentration) / (2 * Tf concentration). | For a given Tf concentration, ↑ iron-saturated Tf may act as a source of host iron for *Mtb* iron capture. | ↑ (versus ↓) % TS. |
| Hemoglobin (Hb) | Hb contains the primary concentration of heme iron, typically accounting for about 65% of total body iron. | Known source of *Mtb*-acquired host iron via *Mtb* heme acquisition system [4.5]. | ↑ (versus ↓) Hb concentrations. |
| Plasma iron (μmol/L) | Serum iron reflects the iron concentration in the blood (versus tissue, cells). | Blood iron concentrations include known, and possibly yet to be identified, sources of *Mtb*-captured host iron. | ↑ (versus ↓) iron concentrations. |
| *Haptoglobin* (Hp) polymorphisms | Hp protein binds free Hb in plasma. | Different Hp polymorphisms are associated with plasma Hp variations including the strength of Hb-binding [7] which may influence the ability of *Mtb* to access this host iron source. | Hp 2-1 + Hp 2-2 (vs Hp 1-1) |
| ***Iron at the level of the Mycobacterium tuberculosis-infected host macrophage*** | | | |
| Plasma ferritin | Plasma ferritin concentrations are directly correlated with macrophage ferritin acting as an iron storage depot. | A known source of *Mtb-*acquired iron [3]. | ↑ (versus ↓) ferritin concentration |
| Plasma soluble transferrin receptor (sTfR) | Plasma sTfR concentrations are directly associated with cellular TfR concentrations, and TfR allows Tf-bound iron to enter body cells. | sTfR concentration will be correlated with the amount of iron entry into cells, and the ability of bacteria to access intracellular host iron. | ↑ (versus ↓) sTfR concentrations |
| Solute carrier family 11 (proton-coupled metal ion transporters),member 1 (*SLC11A1)* polymorphisms | Involved in macrophage-level iron transport. | Alters iron microenvironment at the phagolysosomal level [29] which may influence *Mtb* growth, replication and survival. |  |
|  | SLC1 (rs34448891)  Promoter microsatellite 5 *′(*GT*)*_n_ | Meta-analysis shown to have an association with tuberculosis susceptibility [10]. | Allele 3 (versus Other) |
|  | SLC3 (rs3731865)  Intron 4 single nucleotide transversion:469+14G/C | As above. | C/G, C/C (versus G/G) |
|  | SLC6a (rs17235409)  Nonsynonymous aspartate-to-asparagine change in the carboxy terminal end D543N | As above. | A/G, A/A (versus G/G) |
|  | SLC6b (rs17235416)  3' untranslated region TGTG insertion/deletion:1729+55del4 | As above. | TGTG -/-, TGTG +/- (versus TGTG +/+) |
|  | CAAA (rs17229009)  3' untranslated region CAAA deletion/insertion:276del4 | Shown to have an association with tuberculosis susceptibility [32]. | CAAA -/- (versus CAAA +/-, CAAA +/+) |
