# Supplementary material for: Host iron redistribution as a risk factor for incident tuberculosis in HIV infection: an 11-year retrospective cohort study
Source: BMC Infect Dis. 2013 Jan 29;13:48. doi: 10.1186/1471-2334-13-48 (PMC3568026; doi:10.1186/1471-2334-13-48)
Supplement: Additional file 2 — Baseline characteristics of participants. [file 1471-2334-13-48-S2.docx]

Additional file 2 Baseline characteristics of participants

|  | **Eligible participants** | **Incident tuberculosis** | **No tuberculosis** | ***P**** |
| --- | --- | --- | --- | --- |
| ***Total cohort***, *n* | 1139 | 152 | 987 | - |
| **Demographics** | | | | |
| Age (years), mean ± SD | 34.7 ± 10.3 | 35.7 ± 11.2 | 34.5 ± 10.1 | 0.185 |
| Female, *n* (%) | 649 (57.0) | 75 (49.3) | 574 (58.2) | 0.041 |
| Ethnicity (self-reported), *n* (%) |  |  |  | 0.063 |
| Mandinka | 258 (27.9) | 41 (32.3) | 217 (27.2) |  |
| Jola | 140 (15.1) | 16 (12.6) | 124 (15.5) |  |
| Wolof | 163 (17.6) | 17 (13.4) | 146 (18.3) |  |
| Fula | 128 (13.8) | 26 (20.5) | 102 (12.8) |  |
| Other† | 237 (25.6) | 27 (21.3) | 210 (26.3) |  |
| **Clinical** | | | | |
| HIV status, *n* (%) |  |  |  | 0.688 |
| HIV-1 | 753 (66.1) | 102 (67.1) | 651 (66.0) |  |
| HIV-2 | 371 (32.6) | 47 (30.9) | 324 (32.9) |  |
| HIV-Dual | 15 (1.3) | 3 (1.9) | 12 (1.2) |  |
| Absolute CD4 cells (cells/mm^3^), median (IQR) | 267 (114-513) | 214 (66-413) | 287(121-525) | 0.010 |
| Alpha-_1_-antichymotrypsin (g/L), mean ± SD | 0.47 ± 0.25 | 0.55 ± 0.26 | 0.46 ± 0.24 | 0.001 |
| Body mass index (kg/m^2^), mean ± SD | 20.2 ± 4.1 | 19.3 ± 3.3 | 20.3 ± 4.1 | 0.030 |
| **Iron clinical chemistry, baseline** | | | | |
| Transferrin (g/L), mean ± SD | 1.77 ± 0.60 | 1.57 ± 0.52 | 1.80 ± 0.60 | < 0.001 |
| Transferrin saturation (%),mean ± SD | 32.1 ± 22.8 | 32.5 ± 21.1 | 32.0 ± 23.0 | 0.822 |
| Ferritin (μg/L)‡, median (IQR) | 114 (41-365) | 198 (67-365) | 105 (37-336) | < 0.001 |
| Hemoglobin (g/L), mean ± SD | 10.4 ± 2.2 | 10.1 ± 2.0 | 10.4 ± 2.7 | 0.181 |
| Iron (μmol/L), mean ± SD | 10.3 ± 5.9 | 9.3 ± 4.8 | 10.5 ± 6.0 | 0.043 |
| Transferrin receptor (nmol/L) ‡, mean ± SD | 27.6 ± 16.4 | 28.4 ± 15.6 | 27.5 ± 16.5 | 0.591 |
| **Iron-related genes, polymorphisms and genotypes**^¶^ | | | | |
| *Haptoglobin*§, n (%) |  |  |  | 0.975 |
| Haptoglobin1-1 | 302 (33.4) | 34 (33.7) | 268 (33.4) |  |
| Haptoglobin 2-1 | 438 (48.5) | 48 (47.5) | 390 (48.6) |  |
| Haptoglobin 2-2 | 164 (18.1) | 19 (18.8) | 145 (18.1) |  |
| *SLC11A1,* n (%) |  |  |  |  |
| SLC1 (rs34448891) ^∞^ |  |  |  | 0.681 |
| Allele3/Allele 3 | 639 (69.4) | 83 (70.3) | 556 (69.2) |  |
| Allele 3/Other | 260 (28.2) | 31 (26.3) | 229 (28.5) |  |
| Other/Other | 22 (2.4) | 4 (3.4) | 18 (2.2) |  |
| SLC3 (rs3731865) |  |  |  | 0.337 |
| G/G | 701 (82.4) | 81 (79.4) | 620 (82.8) |  |
| C/G | 143 (16.8) | 19 (18.6) | 124 (16.6) |  |
| C/C | 7 (0.8) | 2 (2.0) | 5 (0.7) |  |
| SLC6a (rs17235409) |  |  |  | 0.620 |
| G/G | 744 (86.8) | 88 (86.3) | 656 (86.9) |  |
| A/G | 107 (12.5) | 14 (13.7) | 93 (12.3) |  |
| A/A | 6 (0.7) | 0 (0.0) | 6 (0.8) |  |
| SLC6b (rs17235416) |  |  |  | 0.748 |
| TGTG +/+ | 577 (68.1) | 76 (71.0) | 501 (67.7) |  |
| TGTG +/- | 240 (28.3) | 27 (25.2) | 213 (28.9) |  |
| TGTG -/- | 30 (3.5) | 4 (3.7) | 26 (3.5) |  |
| CAAA (rs17229009) |  |  |  | 0.568 |
| CAAA -/- | 222 (29.6) | 23 (25.6) | 199 (30.2) |  |
| +/- | 346 (46.2) | 42 (46.7) | 304 (46.1) |  |
| +/+ | 181 (24.2) | 25 (27.8) | 156 (23.7) |  |

*For normally-distributed variables, data are presented as mean ± standard deviation, and statistical significance evaluated using Student’s *t-*test.; for continuous variables that were not normally-distributed, data are presented as the median and interquartile range, and statistical significance evaluated on natural-logarithm transformed data using the Student’s *t-*test; for categorical variables, data are presented as the frequency and percent, and statistical significance tested using the *χ^2^*-test. All tests of significance evaluated the association between participants that did not develop TB against those that did develop incident TB during the study follow-up period.

†Other self-reported ethnicity = Combined Manjango, Serer, Serahuli, Aku.

‡Ferritin concentrations < 2.5 μg/L were imputed with a value of 2.5 μg/L; concentrations >1000 μg/L were imputed with a value of 1000 μg/L; sTfR concentrations < 3 nmol/L were imputed with a value of 3 nmol/L; concentrations >80 were imputed with a value of 80 nmol/L.

§Based on the actual *Haptoglobin* allelic variants measured (Hp^1F^, Hp^1S^, Hp^2FS^) which were then expressed as phenotypes for statistical purposes.

^∞^SLC1 (rs34448891) allele 3 refers to the most common microsatellite allele, referred to as allele 199 in (8).

^¶^Hardy-Weinberg equilibrium test on the total cohort genotype frequencies: *P* > 0.05 = Haptoglobin, SLC1, SLC3, SLC6a, SLC6b; *P* < 0.01 = CAAA.

HIV, human immunodeficiency virus; IQR, interquartile range; n, sample size; SD, standard deviation; TB = tuberculosis (all forms).
